# Advances and Future Challenges in Adenoviral Vector Pharmacology and Targeting

**DOI:** 10.2174/156652311796150363

**Published:** 2011-08

**Authors:** Reeti Khare, Christopher Y Chen, Eric A Weaver, Michael A Barry

**Affiliations:** 1 Virology and Gene Therapy Program, Mayo Graduate School; 2 Department of Medicine, Division of Infectious Diseases, Translational Immunovirology and Biodefense Program; 3 Department of Molecular Medicine, Department of Immunology, Mayo Clinic, Rochester, MN 55905, USA

**Keywords:** Ad serotypes, liver, sequestration, serotypes, targeting.

## Abstract

Adenovirus is a robust vector for therapeutic applications, but its use is limited by our understanding of its complex *in vivo* pharmacology. In this review we describe the necessity of identifying its natural, widespread, and multifaceted interactions with the host since this information will be crucial for efficiently redirecting virus into target cells. In the rational design of vectors, the notion of overcoming a sequence of viral “sinks” must be combined with re-targeting to target populations with capsid as well as shielding the vectors from pre-existing or toxic immune responses. It must also be noted that most known adenoviral pharmacology is deduced from the most commonly used serotypes, Ad5 and Ad2. However, these serotypes may not represent all adenoviruses, and may not even represent the most useful vectors for all purposes. Chimeras between Ad serotypes may become useful in engineering vectors that can selectively evade substantial viral traps, such as Kupffer cells, while retaining the robust qualities of Ad5. Similarly, vectorizing other Ad serotypes may become useful in avoiding immunity against Ad5 altogether. Taken together, this research on basic adenovirus biology will be necessary in developing vectors that interact more strategically with the host for the most optimal therapeutic effect.

## INTRODUCTION

**Fig. (1):**
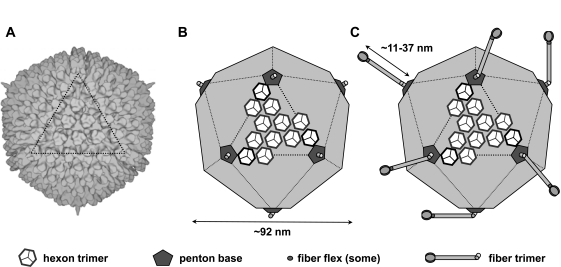
**Schematic of Ad capsid structure.** (a) Cryo-electron micrograph reconstruction of the Ad5 capsid. The dotted triangle overlays one of the 20 facets of the icosahedron. (b) Diagram of the adenoviral capsid showing a "group of nine" hexon trimers, penton bases, and fiber n-terminus that is observed in cryo-EM. (c) Addition of flexible fiber structures to B that are not observed in cryo-EM.

Adenoviruses (Ads) are icosahedral non-enveloped DNA viruses with diameters approximately 90 to 100 nm (Fig. **1**). Ads were first isolated from human adenoids in 1953 and since then, bovine, ovine, murine, canine, equine, porcine, and caprine specific strains have also been identified [1]. With over 50 currently recognized human serotypes, Ads are widely present in human populations (Table **1**). These viruses are typically associated with mild disease, however more severe complications may occur in infants or in immunocompromised patients. Most Ad serotypes manifest with mild respiratory symptoms, although others have various pathologies like acute respiratory disease (Ad3, 4, 7, 14, 21), keratoconjunctivitis (Ad8, 9, 10, 19), gasteroenteritis (Ad40, 41), and even obesity (Ad36) [2-4].

Adenoviruses have several features that make them inherently useful as oncolytic, vaccine, or gene therapy vectors. For instance, they are non-enveloped viruses and are therefore sufficiently stable for packaging as lyophilized preparations in vials or capsules, even without a cold chain. They mediate high transduction efficiency in non-dividing (i.e. most human somatic cells) and dividing cells (i.e. transformed cells) and can generate 10^4^ virus particles (vp) per infected cell. This supports large-scale preparations of 10^13^ vp from 10^9^ cells. 

Ad genomes range from ~36-40 kilobase pairs (kb) in length and can carry large transgenes up to this size. Their DNA genome and high fidelity DNA polymerase confers relative genomic stability in comparison to RNA viruses. Furthermore, unlike retroviruses, Ad genomes are non-integrating. While this poses minimal risk for insertional mutagenesis [5] these vectors are comparatively ineffective for the genetic modification of dividing cells since cell division will result in loss of the transgene [6, 7]. On the other hand, Ad genomes can persist for years in non-dividing cells provided that an immune response is not produced against Ad or the transgene product. 

When applied as a gene therapy agent, intravenous delivery of Ad5 into mice produces supraphysiologic levels of its transgene. For example, *in vivo *genetic modification of mice with Ad5 resulted in 6 mg/ml of α1-antitrypsin in the circulation; these are levels at which the transgene became the second most abundant protein in the blood [8]. In other words, when Ad has been applied as a gene-based vaccine it is one of the most robust platforms. 

While Ads are potent *in vivo *gene delivery platforms, they are also robust at generating immune responses. For example, a head to head comparison with vaccinia virus vectors or plasmid DNA vaccines in non-human primates demonstrated that Ad mediated the most robust immune responses [9]. This effect is fortuitous for vaccine purposes, but is problematic for gene therapy approaches. For instance, pre-existing immunity against the virus or the transgene protein reduces the persistence of genetic correction [5-7, 10, 11]. 

This review is an update and expansion of a previous review by our laboratory that was published in 2007 ([12] http://www.ncbi.nlm.nih.gov/pmc/articles/PMC2244792/). This earlier review provided a comprehensive discussion of Ad protein composition, structure, and life cycle. It also integrated applications of basic Ad biology in terms of vector targeting and strategies for vector improvement, such molecular linkers, genetic, and chemical modifications with a particular emphasis on modification of the Ad fiber protein for targeting efforts. The 2007 review was also Ad5-centric, focusing on the most utilized adenoviral serotype for gene therapy.

In this update, we have incorporated recent information of Ad vector biology and targeting as it stands in 2011. In particular, we address the growing understanding of *in vivo* virus pharmacology rather than *in vitro* virus-cell biology. We discuss the biology of Ad5 and other Ad serotypes *in vivo* with particular emphasis on the known and speculated pharmacology “sinks” for these viruses that affect their use for systemic or targeted therapy. This review will also discuss recent observations regarding the role of the Ad hexon protein on *in vivo *(but not *in vitro*) virus tropism and how recent engineering efforts have been directed more at adenovirus “detargeting” than adenovirus “retargeting”. With detargeting of significant pharmacologic sinks nearly at hand, the field will now likely be able to apply the retargeting strategies that appeared promising *in vitro*, but that have historically failed *in vivo*.

## ADENOVIRUS SPECIES AND SEROTYPES

**Fig. (2):**
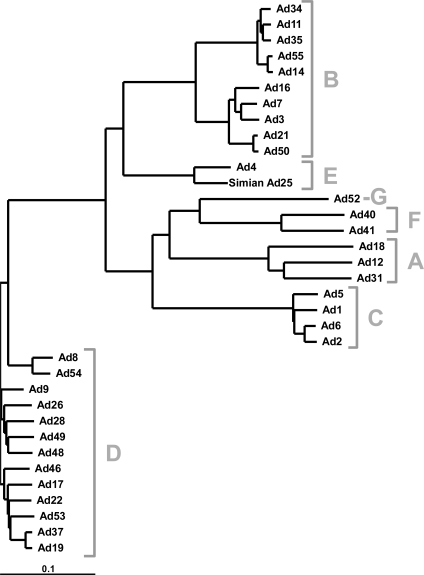
**Phylogenetic Tree of Human Adenoviruses.** Full genome comparison of 34 completed Ad sequences groups viruses with species grouping according to genetic similarity.

Adenoviruses were originally defined by a number of bioassays including cross-susceptibility to neutralizing antibodies and subsequent categorization into serotypes. With the advent of DNA sequencing, newer viruses are now being characterized by genotype and phylogenetic comparisons to other Ad genomes (Fig. **2** and see [13] for an excellent review). Current convention is to describe distinct Ads as new serotypes although they are now typically classified by genotyping. 

Based on serotyping, related human and non-human Ads were formerly referred to as subgroups. With the advent of more sequence data, this designation has been revised to describe the different groups as species. There are currently 55 human adenovirus serotypes that distribute into seven species from A to G (Table **1**) [14]. As of this writing only 34 full genome sequences are available for full genome comparison (Fig. **2**). The vast majority of data on the biology of Ads has been garnered using species C adenoviruses Ad2 and Ad5 in cell culture. Therefore, most knowledge of virus-cell interactions is based largely on two out of now 55 human Ad serotypes. While many lessons learned with Ad2 and Ad5 will still apply to other Ads, many others do not apply.

More recently, a variety of groups have delved into the biologies of other human and non-human Ads in the quest for new functionalities or to evade anti-Ad5 immunity in patients [15-23]. In this review, we describe some aspects of novel Ad biologies that vary between species and serotypes. For additional information, see several reviews on different Ad serotypes [10, 24-26].

## NATURAL CELL BINDING AND ENTRY BY ADENOVIRUSES

*In vitro,* adenoviruses infect permissive host cells rapidly and efficiently. Typical time from infection to the production of new virions ranges from 14 to 19 hours depending on serotype [27]. Initial interactions with cellular receptors could theoretically be mediated by any surface exposed protein on the virus (Table **2** and see [12] http://www.ncbi.nlm.nih.gov/ pmc/articles/PMC2244792/) for background on virus structure/function). Early work in Ad interactions identified a subset of proteins that interact with receptors *in vitro* (fiber, penton base) and *in vivo *fiber, penton base, hexon, (Fig. **1**). As more of these interactions are revealed exposed, more proteins will likely be found to interact directly or indirectly with receptors and proteins under certain circumstances (see interactions of hexon with blood factors below). 

### Adenovirus Major Capsid Proteins

There are three major capsid proteins on adenoviruses: fiber, penton base, and hexon (Fig. **1**). There are 36 monomers of fiber, 60 monomers of penton base, and 720 monomers of hexon on each Ad virion. There is good evidence that the fiber and penton base proteins of many Ad serotypes interact directly with cellular receptors. In contrast, there is little evidence showing that hexons display a ligand for cellular receptors. The massive number of hexons per virion certainly has the possibility of mediating avidity interactions via multivalent charge interactions. However, a high affinity evolved ligand has not been associated with Ad5 or any Ad hexon to date. 

Under the simplest circumstances on permissive cells, the Ad fiber protein acts as the primary high affinity attachment protein for the virus *in vitro*, provided its receptor is expressed on cells. Three fiber monomers trimerize to form an antenna-like structure located at each vertex of the icosahedral capsid (Fig. **1C**). The tail domain of the trimer attaches directly to penton; the shaft length is determined by multiple amino acid repeats; the knob domain confers specificity to cellular receptors.

Although fibers across the seven human Ad species have the same basic structure, their amino acid sequences and shaft lengths vary considerably. Prototype Ad5 fiber binds to CAR, the coxsackie and adenovirus receptor (reviewed in [12, 25]). Viruses from other species can bind to CAR, CD46, sialic acid, desmoglein-2, and perhaps other receptors [25, 28].

*In vitro, *Ad5 binds CAR and also binds to cellular α_v_ integrins [29]. Ad5 binds CAR with 15 nM affinity, whereas penton base engages integrins with 10-fold lower affinity [29]. Because of this affinity difference, species C viruses have been shown to first engage CAR and then rapidly transition to binding and entry via interaction with integrins [30]. This dual receptor utilization is made possible by the length and flexibility of the Ad5 fiber (Fig. **1C**). Ad5 has one of the longest fiber proteins with 21 β-spiral repeats in its shaft [31] (sometimes referred to as having 22 repeats [25]). 

Exactly how Ad5 could transition from binding CAR to binding integrins was unclear until it was realized that its fiber is able to bend due to a flexible lysine-lysine-threonine-lysine (KKTK) motif at repeat three in its shaft ([32] and Fig **1C**). This flexibility can be appreciated in cryo-electron microscopic (cryo-EM) reconstructions of Ad5 for although they are one-third the diameter of the icosahedron, the twelve 35 nm fibers are not observed. Instead, only a stump of the fiber can be seen (Fig. **1A**) [33, 34]. This loss of fiber electron density results from the computational assembly and integration of hundreds of the images of individual Ad virions during cryo-EM image reconstruction. Proteins like hexon, that are fixed in space on the virion resolve into structures (Fig. **1A**). If the proteins are flexible, then they will be in a different orientation with respect to the virion. When this is averaged between hundreds of virions, these mobile Ad5 fiber proteins disappear. In contrast, when short-shafted fibers that lack this flexible KKTK domain are imaged, their shafts can be observed [35]. Fibers also have an additional flexibility motif between the fiber shaft and knob domain ([31] and Fig. **1C**). This can be inferred in cryo-EM using short-shafted Ad35 fiber, since its shaft is observed, but its knob is not [35]. 

This flexibility allows the long-limbed Ad5 to undergo "virus yoga" [32] wherein the fiber knob binds CAR and then the shaft flexes to allow an arginine-glycine-aspartic acid (RGD) motif on penton to bind to α_v_β_1_, α_v_β_3_, α_v_β_5_ or α_3_β_1 _integrins on the cell surface [29]. Binding of RGD to integrins triggers Ad5 internalization via receptor-mediated endocytosis on clathrin-coated pits. The virions are subsequently able to escape from endosomes and traffic to the nucleus within 30 to 60 minutes of cell binding on permissive cells (reviewed in [12]). If the fiber receptor is absent or if CAR binding is ablated on knob, the virus can bind more slowly by lower affinity interaction of penton base with integrins. In this circumstance *in vitro, *short (1 hour) exposure of CAR-negative cells with Ad5 results in poor transduction whereas longer (24 hour exposure) can mediate very high transduction (e.g. Ad5 on K562 cells [36] and data not shown). If cells lack CAR and integrins, they are relatively (but not absolutely) resistant to Ad5 infection. 

This *in vitro *infection paradigm is based on Ad5 and Ad2 infection in cell culture. These rules apply to some extent to other Ad species and serotypes with some minimal to drastic variations. For example, other species C Ads behave similarly to Ad5* in vitro*, although the fiber of Ad6 is three repeats shorter – perhaps making its yoga process somewhat less efficient [37]. In contrast, most other species of human Ads have markedly shorter fibers than Ad5 (with only 6 or 8 shaft repeats) creating a disconnect between being flexible enough to bind a receptor and being able to use a receptor for cell infection. These shorter shafted fibers may target receptors that are directly endocytosed such that integrin interactions are less important.

For example, the fiber for species D Ad37 can bind to CAR, CD46, and sialic acid. However, it appears only able to use sialic acid as a receptor for infection [18, 38, 39]. This effect may be due to the very short length of species D fibers with only 8 β-spiral repeats. These fibers may lack sufficient flexibility or length to allow functional combination receptor utilization such as Ad5 can perform between CAR and integrins. Similar effects are observed with short species B viruses Ad35 that have fibers with only 5.5 repeats. These viruses are able to use CD46 or other receptors efficiently with these shorter shafts [40]. However, if CAR-utilizing viruses are given short shafts, this abrogates effective combination of CAR and integrin and infectivity is markedly reduced. 

Short-shafted fibers may also enable Ads to use integrins more efficiently as primary receptors. For example, comparison of species D Ad8, 9, 19, 19a, and 37 demonstrated that Ad8, 19a, and 37 use sialic acid as a functional receptor, whereas Ad9 and19 appeared to use α_v_ integrins as their primary receptor *in vitro *[38]. In our recent screen of species D Ads for infection of B cell cancers, we observed that most were largely independent of sialic acid for infection, but instead used a combination of CD46 and α_v_ integrins for infection and killing [41]. Therefore, longer fibers appear to create more steric hindrance with penton-integrin interactions and may require more flexibility. In contrast, shorter shafted fibers may obstruct penton-integrin interactions to a lesser extent and better use this infection pathway on cells. 

## ADENOVIRUS PHARMACOLOGY *IN VIVO*

### Interactions with Blood Factors Impart Tropism

Given that both fiber and penton have receptor-binding motifs, viruses with inactivating mutations were tested for "detargeting"[42]. While mutations to ablate CAR and integrin binding worked as expected *in vitro, *they had surprisingly weak effects on *in vivo *tropism [43, 44]. Subsequent seminal work suggested that the difficulty in altering the tropism of Ad5 was actually due to unexpected interactions of the virus with host proteins *in vivo *[45]. Initial studies indicated that Ad fiber can bind blood factors like FIX and C4BP with moderate affinity [45]. However, later studies independently concluded that hexon, not fiber, was the main protein interacting with blood factors [46-48]. These studies showed that factor X (FX) binds Ad5 hexon with nanomolar affinity and that FX then targeted the virus to receptors on hepatocytes. Comparison of FX binding to select Ads from different Ad species demonstrated that approximately half of the tested viruses bind FX [48]. 

Blood factors I to XIII and Protein C (PC) are produced in the liver as zymogens and are activated by cleavage for use in normal blood clotting. Vitamin K dependent blood factors VII, IX, X and protein C enhance transduction of Ad5 *in vitro* [45, 46]. These factors share the common domain structure GLA-EGF1-EGF2-SP, where SP is the catalytic serine protease domain, EGF1 and EGF2 are epidermal growth factor-like domains, and the GLA domain is a hexon-binding glutamate rich domain [46, 47, 49, 50]. On the other hand, non-homologous factors (FXI and FXII) do not enhance transduction [46]. Upon production in the liver, zymogens containing the GLA domain are carboxylated in a vitamin K dependent manner on the γ-carbon of each of their 9-12 glutamic acid residues [24]. *In vivo*, physiologic levels (8-10 μg/ml) of these γ-carboxylated blood factors results in hepatocyte transduction even for CAR-ablated viruses. Conversely, inhibition of this post-translational vitamin K dependent γ-carboxylation with warfarin markedly decreases hepatocyte transduction by Ad5 [46]. FX binding to hexon is highly calcium dependent and can be inhibited by chelation with EDTA. This is unsurprising, as the GLA domain binds seven calcium ions, while the EGF1 and SP domains each bind one. As a result, the absence of the GLA domain renders Ad5 unable to bind FX (as measured by surface Plasmon resonance) and thus unable to transduce hepatocytes [47, 48].

Binding of the GLA domain of FX to Ad virions appears to orient the SP domain of FX to bind heparan sulfate proteoglycans (HSPGs) on cells. After i.v. injection, this appears to yield selective infection of hepatocytes. However, HSPGs are ubiquitously found on the cell surface and on extracellular matrix proteins, so the mechanism of specific uptake into hepatocytes is unclear. Recently, Bradshaw *et al.* showed evidence that a high level of N- or O-sulfation (“sulfation signature”) on heparan sulfate in the liver is necessary for FX mediated Ad infection, thus accounting for liver specificity [51].  In addition, the fenestrated vasculature of the liver provides easy access to hepatocytes as opposed to other tissues where these “windows” are not present.

Ads display seven hypervariable region (HVR) domains on their hexon proteins that vary in sequence between serotypes [52] and are primary targets for neutralizing antibodies [53, 54]. Variations in these HVRs appear to correlate with FX binding affinity and with the ability of Ad serotypes to transduce hepatocytes [47]. Cryo-electron microscopy of Ad5 identified FX density near the central depression of the hexon trimers near the HVRs [47, 48]. Mutational analyses of Ad5 hexon suggested interactions of FX with HVR5 and 7 [55]. More precisely, a point mutation of glutamic acid 451 that is conserved in FX binders partially abolished binding of FX to the virus [55]. 

These data suggest roles for HVR5 and 7 in FX binding. However, even within Ad5's own species C viruses, there is marked variation in liver transduction; Ad5 and Ad6 are most robust and Ad1 and Ad2 are markedly less effective [37]. When the HVRs of these viruses are compared, only the HVR1 and 4 genotypes cluster with the higher liver transduction phenotype. This is interesting given that the HVR1s of Ad5 and Ad6 have considerable negative charge (net negative charge of 13 and 8). At the same time, the GLA domain of FX displays 7 or more Ca^2+^ and ions, it is interesting to speculate that binding or perhaps initial docking of FX with hexon may be facilitated by FX interaction with HVR1. Given that HVR1 is unstructured in x-ray crystals, interactions with this loop cannot be modeled easily. Therefore, targeted mutation of HVR1 has not yet been tested. 

These observations induced a paradigm shift in Ad biology by demonstrating that *in vivo *tropism of some Ads is mediated not just by ligands on the virus, but also by host factors. This showed that Ad5 is an excellent choice of vector for liver-directed gene therapy as it has natural tropism to the liver upon systemic injection and can transduce hepatocytes with high efficiency. Conversely, Ad5 may be a poor choice for therapy beyond the liver, since the bulk of the virus is depleted in the liver. Whatever the intended target tissue, a deeper understanding of Ad pharmacology reveals that host sequestration mechanisms can result in only a fraction of the injected dose reaching its intended location. Here we outline the obstacles that Ad encounters *in vivo *in order to further inform the optimal design of Ad vectors. 

### Interactions with Blood Cells and Proteins

Upon intravenous delivery, evidence suggests that Ad5 interacts with a number of soluble proteins including natural antibodies, complement [56] and blood clotting factors [45-48]. These adsorptions likely occur immediately after virus injection into the blood stream. Natural antibodies are circulating antibodies not induced by individual antigens, but encoded by the germline against common structures. Consequently, they are broadly specific, tend to have low affinity, and provide naive hosts immediate defense against invading pathogens like bacteria and viruses [57]. Natural antibodies somewhat compensate for their low affinity with high avidity and are predominantly IgM. Thus, natural antibodies also serve as a potent complement activator, although Ad5 can also bind directly to complement proteins from the classical and mannan-binding lectin pathways [56, 58]. 

Studies have observed binding of complement protein C3 to the Ad capsid in the presence of factor B and factor D, proteins involved in the antibody-independent alternative pathway of complement activation [59, 60]. Interestingly, complement protein C1q was shown to increase transduction in CAR negative cells [61]. On the other hand, complement binding to adenovirus is immunostimulatory, and can been reduced by incorporating a complement-binding inhibitory peptide into fiber or pIX of the viral capsid [62]. Notably, the mechanisms of complement activation *in vivo* have been shown to be different than the mechanisms derived *in vitro*. *In vitro,* antibodies are required for C3a binding and subsequent activation of the classical complement pathway. *In vivo*, antibodies are not required for C3a binding, and activation can occur through both classical and non-classical pathways [58].

Complement binding can lead to clearance of the virus via macrophage uptake, but recently it was also shown to be involved with sequestration of the virus on erythrocytes. Carlisle *et al.* found that Ad5 appeared to interact with complement factors which then act as bridge for binding to human complement receptor 1 (hCR1) on erythrocytes [63]. Murine erythrocytes displaying hCR1 significantly decreased the amount of Ad5 able to transduce the liver *in vivo* (16 fold). Furthermore, CAR is also displayed on human, but not murine, erythrocytes despite its role as a cell adhesion molecule. As erythrocytes are not productively transduced, they can therefore act as a substantial sink for any systemically-administered Ad [64]. 

In addition to human erythrocytes, the majority of human thrombocytes (72%) are also positive for CAR [65]. Although CAR has not been tested as the specific mediator for the interaction, platelet pull downs and transmission electron microscopy reveal that Ad5 binds directly to thrombocytes and activates them [66]. The von Willebrand factor, one of the proteins released during platelet degranulation, is implicated in causing platelet-leukocyte aggregates as well as the widely observed phenotype of Ad5 induced thrombocytopenia [65].

### Systemic Distribution of Adenoviruses

**Fig. (3):**
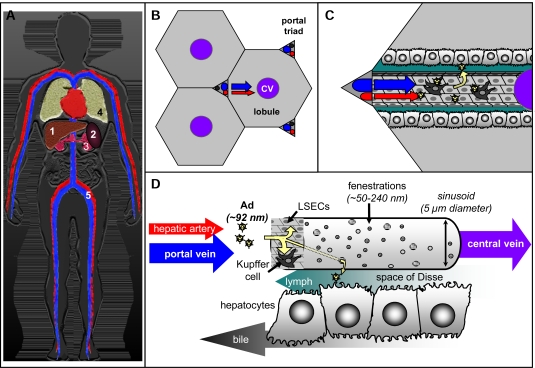
**Adenovirus delivery upon systemic injection.** (a) Distribution of virion delivery including largest pharmacologic “sinks”. 1 = Liver, 2 = Spleen, 3 = Kidney, 4 = Lung, 5 = Bloodstream. Schematic of viral migration from the blood stream into the parenchyma of liver: (b) within the lobule structures of the liver and (c,d) within one lobule. (c) represents flow of virus from the triad to the central vein looking down into the sinusoid. (d) represents permeation of virus from inside the sinusoid out into the parenchyma via fenestrations. (d) also shows other pharmacologic paths virions may take including outflow in the the lymph and bile.

When delivered i.v. directly into the bloodstream, Ads will be delivered to the heart via the inferior or posterior vena cava, be pumped through the lungs, and then be sent to the periphery via the aorta and arteries (Fig. **3A**). Free or bound virus can circulate through the body to encounter any cell or tissue in contact with the blood. An intravenous (i.v.) dose of Ad will likely first encounter the heart and lungs before being distributed to the liver, spleen, and kidneys (Fig. **3A**). Furthermore, general endothelial cells lining the vasculature throughout the body may theoretically be a huge sink for any Ad serotype. While there is evidence that Ad5 and other serotypes infect endothelial cells, these interactions do not appear to be particularly productive, perhaps due to low levels of cognate receptors [67, 68]. To what degree bulk endothelial cells differ from liver sinusoidal endothelial cells (LSECs) in Ad interactions is unclear. Obvious differences relate to the ability LSECs to form fenestrations and to pinocytose material (see below). 

The body eliminates most compounds, including Ads, through the natural filtering functions of the liver and kidneys. Water-soluble items can be removed by the kidney and hydrophobic compounds are generally metabolized in the liver for subsequent excretion in the gut or in the kidney. While Ads can and do accumulate in a variety of organs, the liver appears to be the principal sink for prototype Ad5 virus. This filter function is demonstrated in elegant pharmacologic studies in mice. At doses up to 4x10^12^ vp/kg of Ad5, approximately 98% of injected virus is found in the liver 30 minutes after injection [69]. At this same dose, only about 1% of injected Ad5 can be found in either the lungs or the kidney at this dose. If the dose is increased 4-fold, Ad5 in the liver falls to 85% of injected dose and virus in the spleen and lung rises to 6 and 5% of injected dose, respectively. 

It should be noted that when normalized to organ weight (i.e. specific activity instead of total activity) the spleen can appear to express Ad5 nearly as well as the liver [70]. This representation is helpful for understanding adenoviral biology, but may minimize the true effect of the virus *in vivo* since the liver is substantially larger. Such analyses become appropriate in terms of generating immune responses to Ad or its transgene products, since total expression of the immunogenic epitopes is more relevant than specific activity 

## VECTOR SEQUESTRATION IN THE LIVER

Liver sequestration is remarkably fast as we demonstrated recently using near-infrared (NIR) fluorescent imaging in mice [71]. Ad5 was labeled with the NIR fluorophore IR800 and injected i.v. into the jugular vein. By fast image capture (250 millisecond exposures every 0.5 millisecond), virus could be seen entering the heart within 500 milliseconds of injection. Within 7 seconds, the viral swarm was observed in arterial outflow throughout the mouse. Virus then returned from the periphery and began accumulating in the liver. Within 3 minutes of injection, the bulk of virus distribution was essentially complete with the vast majority being localized to the liver and less so to the spleen and kidneys [71]. 

In humans, approximately 1.5 liters of blood is delivered into liver sinusoids from the portal vein and hepatic artery every minute for filtration (Fig. **3B**, **C**, and **D**). Particulates like Ad that enter liver lobules and their sinusoids encounter Kupffer cell macrophages and LSECs that serve as gatekeepers for the liver (Fig. **3C** and **D**). There is some evidence that Ad5 that is ensnared on platelets, is trafficked to the liver, and also likely trafficked to the spleen. Indeed, thrombocytes may deliver viral particles to resident liver macrophages, also known as Kupffer cells, within minutes of intravenous injection. Depletion of platelets prior to adenovirus injection in one model was able to decrease the amount of viral DNA in the liver [66]. In contrast, blood factor binding or platelet binding to Ad5 was not found to contribute to Kupffer cell uptake in other studies [56]. Therefore, it is somewhat unclear whether platelets play a role in delivery of Ad5 and other serotypes to liver Kupffer cells.

Viruses that escape both Kupffer cells and LSECs can enter the space of Disse through fenestrations in the LSECs (Fig. **3C** and **D**). Once in the parenchyma, virions can interact with hepatocytes via evolved or captured cell binding ligands to mediate transduction or liver damage depending on serotype. Viruses that fail to infect hepatocytes after entry into the space of Disse are presumably captured in the lymph from the lobules and delivered to draining lymph nodes (Fig. **3D**). If viruses are transcytosed (perhaps by engaging caveolin rather than clathrin entry pathways) they could theoretically be ejected on the other side of the hepatocytes into the bile (Fig. **3D**). The effects or level of virus distribution, transduction, and infection from the lymph and bile of the liver remain largely unexplored. Given their likely delivery to mucosal and immune cell education sites, these routes of virus distribution may have profound effects on immune responses against Ads and their transgene products. 

### Kupffer Cells

Kupffer cells (KCs) are the resident macrophage of the liver and are essential in removing foreign particles and pathogens from the blood stream (Fig. **3C** and **D**). Although they comprise only ~7% of liver cells, they estimated to account for 80-90% of all of the macrophages in the body [72, 73]. It has been estimated that liver Kupffer cells can sequester up to 98% of intravenously injected Ad5 vector in mice [74]. These interactions are thought to be predominantly phagocytic and non-productive for infection, since this uptake triggers massive destruction of virions and the Kupffer cells [74, 75]. However, a small proportion of virions that interact with Kupffer cells may enter by integrin binding to mediate low level transduction of these cells [76]. For example, at very high doses of Ad5 (1 x 10^11^ PFU/kg), 70% of hepatocytes and 15% of Kupffer cells expressed transgene three days later [76]. 

Scavenger receptor A (SR-A) is a broadly specific scavenger receptor expressed on the surface of Kupffer cells that is thought to recognize net negative or positive charge [56]. Injection of negatively charged polyinosinic acid, or poly(I), into mice prior to injection of Ad5 transiently increased viral circulation in the blood 10-fold; transgene expression increased in a variety of tissues by 5- to 15-fold [77]. The hypervariable region 1 (HVR1) on Ad5 hexon in particular has large amounts of charged residues and has therefore been implicated in Kupffer cell recognition [74]. On the other hand, Kupffer cells in wild-type and SR-A knock out mice appeared to take up similar amounts of Ad5, suggesting that Kupffer cells have alternate or redundant mechanisms for viral recognition [68]. 

### Liver Sinusoidal Endothelial Cells (LSECs)

Liver sinusoidal endothelial cells (LSECs) are also a major component of the reticuloendothelial system (RES) although their role in phagocytosis of particles like Ad is underappreciated [78]. LSECs line the sinusoids of the liver and represent ~25% of liver cells ([72] and Fig. **3C** and **D**). Like Kupffer cells, LSECs express scavenger receptors SREC 1 and SREC-II that may be candidates for uptake of Ad particles [79]. LSECs work in concert with Kupffer cells to clear material from the bloodstream. Unlike Kupffer cells that can engulf particles up to 2 µm in diameter, LSECs remove particles under 230 nm in diameter by pinocytosis [78, 80]. Therefore, both cell types of have an overlapping ability to remove Ads from the circulation, although an increase in the effective diameter of Ad virions upon binding to other circulatory factors may favor uptake by Kupffer cells. Interestingly, when Kupffer cell uptake was impaired, LSECs were able to take up particles >1 µm in diameter [80]. Sequestration of Ad by vascular endothelial cells is unclear, although *in situ* experiments show that like LSECs they are poorly transduced even at high doses [81]. 

Like Kupffer cells, LSECs are inefficiently transduced by Ad5, showing no expression at <100 infectious units (IU)/cell, and meager expression at >1000 IU/cell *in vitro*. In contrast, 100% of hepatocytes are infected by Ad5 at 5 IU/cell. Similar results have been observed *in vivo. *For example in mice, no transduction of Kupffer cells or LSECs was observed after injection of Ad5 [82]. 

### The Effects of Host Species and Strains on Adenovirus Pharmacology 

The complexity and crucial differences in uptake of Ad5 by the liver is reflected in direct comparisons of transgene expression in various inbred mouse strains. At a moderate dose of 1 x 10^10 ^vp Tao *et al.* demonstrated a ~400-fold range of variation in Ad5 liver transduction in NCR nude, C57Bl/6, BALB/c, C3H, and Rag-1 mice. Higher doses of 8 x 10^10 ^vp, which test expression effects after exceeding the Kupffer cell “threshold” show a different profile of expression in these mice, with a 30-fold range in variability [83]. Snoeys *et al.* determined that BALB/c mice take up ~6-fold more Ad DNA in their non-parenchymal cells than C57BL/6 mice. When analyzed by cell type, they showed that BALB/c mice sequestered ~20 times more Ad DNA in their Kupffer cells than C57BL/6 mice after i.v. injection [84]. Conversely, C57BL/6 mice took up more virus in their LSECs rather than in their Kupffer cells. Interestingly, they found that the overall number of Kupffer cells between the two mouse strains is not significantly different, suggesting that fundamental differences in the location, types, and/or density of receptors responsible for Ad uptake may be significantly disparate between animal strains.

Electron microscopy shows that LSECs form sieve plates with fenestrations [85, 86] that allow Ads to reach the parenchyma of the liver and hepatocytes [87, 88] (Fig. **3**). However, fenestration sizes can be highly variable between species: >150 nm for Sprague Dawley rats, 141 nm for C57Bl/6 mice, 103-105 nm for two strains of rabbits and 107 nm for healthy human livers [72, 89]. 

These data have been used to propose that the effect of Ad in humans may not be predicted by murine models. While this may be the case, it should be noted that these fenestration measurements are *mean* sizes, not an absolute cut-off. For example, the mean fenestration size in healthy human livers were reported as 107 nm although diameters may reach up to 240 nm in diameter [72, 89]. Indeed, in human liver, more than half of the measured fenestrations exceeded the diameter of Ad. These theoretical calculations are supported by human data. For instance, increases in liver enzymes are routinely observed when human patients have been injected with replication-competent, replication-selective, or replication-defective Ad5 by intravascular injection or even after intratumoral injection [90-92], suggesting that virus must be reaching hepatocytes in order to stimulate such release. 

Our original tests of Ad pharmacology were performed in outbred mice to avoid the effects of inbreeding in favored mouse strains [67, 70, 88]. As we have moved to evaluating immune responses to Ad and its transgenes and utilizing genetically modified mice, we have transitioned to working in inbred strains or on different inbred genetic backgrounds [23, 93-96]. We have found that the genetic background of the mice can have profound effects on the pharmacology of Ad as evidenced by recent work in our laboratory. Comparisons of BALB/c, C57BL/6, FVB, 129, nude, and hairless HRS mice demonstrate considerable differences in raw gene expression in the liver after intravenous injection and after manipulating the levels of Kupffer cells prior to injection ([94, 95] and unpublished data). The use of different strains of mice may explain some diametrically-opposed results in the literature particularly regarding the effects of Kupffer cell depletion and why certain strains of mice will respond to gene therapy and others will not. Until the key biologies are identified that control these pharmacologic effects and how they apply in humans, the best approach is to test Ad pharmacology in several strains and in other species.

### Beyond the Liver

The discussion above has focused on what we currently consider the “biggest” initial steps and barriers that adenoviruses encounter after an intravenous injection. Beyond the blood, vascular endothelial cells, and the liver Big Three (i.e. Kupffer cells, LSECs, hepatocytes), there are of course other cells within the liver that likely encounter and interact with adenoviruses (i.e. liver dendritic cells, stellate cells, lymphocytes, etc.). Beyond the liver, we know that a smaller, but significant fraction of Ad lands in the spleen, kidneys, and lungs after intravenous injection (Fig. **3A**). Indeed, different serotypes of Ad are likely to permeate to many sites that we currently do not have the sensitivity to track (other organs, tissues, lymphatics, etc.). These locations also impact the pharmacology and, importantly, immune responses against Ads and their transgenes. 

## ADENOVIRAL VECTOR DETARGETING

The vast majority of early work to modify Ad tropism was directed at retargeting the virus to new receptors. Subsequent work aimed to detarget Ad from its cognate *in vitro* receptors. When these efforts have been applied *in vivo,* they have generally failed for lack of decent targeting ligands and because of limited understanding of Ad pharmacology in an intact body. The revolutionary observations concerning the depletion of Kupffer cells by Ad and the unexpected effect of clotting factors on Ad pharmacology have opened up a new area to apply effective detargeting strategies. We hypothesize that once we can detarget the wrong cells effectively *in vivo*, then retargeting with new ligands may begin to succeed. However, we may need to know all of the cells and proteins that are mistargeted before detargeting will be optimal. 

### Evading Blood Proteins and Cells

After intravenous injection, Ads bind proteins and cells in the blood. As some of these interactions are ligand-receptor driven (i.e. hCAR and hCR1 receptors on erythrocytes, etc.) the use of alternate serotypes may attenuate some effects. Other approaches are to genetically-delete these evolved viral ligands to specifically detarget CAR, CD46, integrin, and other interactions [68]. 

Another approach to evade interactions is to coat Ads with linear or looped hydrophilic polymers like polyethylene glycol (PEG) and poly-*N*-(2-hydroxypropyl) methacrylamide (HPMA) ([64, 67, 69, 70, 74, 88, 93, 97-111] and see below).

Polyethylene glycol (PEG) is a stable, uncharged, hydrophilic, and synthetic polymer widely used in food and drug industries. Made up of repeating units of ethylene oxides (CH2-CH2-O-) it can be synthesized in multiple shapes (e.g. branched and linear) and varying lengths (resulting in molecules up to 40 kDa in size). PEG can be manufactured with functional groups on their one or both of their termini, resulting in its use as a versatile chemical modifier for adenovirus. Heterobifunctional PEGs have functional groups on either end of the molecule. This enables its use as a linker molecule, or can be useful in detection of PEGylated virions. For instance, heterobifunctional PEG such as Alexa488-PEG-maleimide can be covalently linked to cysteines on the capsid of Ad5, thus tagging it with a fluorescent marker [112].

HPMA is a hydrophilic polymer composed of an unreactive carbon chain backbone with diglycyl side chains. Approximately 10 amino-reactive 4-nitrophenoxy groups are incorporated into these side chains per 100 monomers of the backbone. Thus, HPMA is a multivalent polymer and can interact with an Ad capsid at multiple locations like a zipper. 

Both of these polymer approaches prevent interactions of Ad with a variety of blood proteins and cells. For example, PEGylation of Ad5 blocks CAR binding, thereby eliminating interactions with any CAR-expressing cell [70]. For example, PEGylation and/or HPMA modification blocks binding and activation of platelets, red blood cells, and endothelial cells by Ad5 [64, 67, 113]. These effects in the blood or tissues have substantial abilities to reduce innate immune responses and liver damage after i.v. injection of Ad. However, random polymer modification can inhibit virus function [70]. Therefore, approaches to target PEGylation to specific sites on Ad by using maleimide-PEG to react with inserted cysteines may reduce interactions with blood cells while preserving virus activity [95, 105, 106, 114].

### Evading the Liver: Kupffer Cells

In mice, 98% of a low dose of Ad5 is found in the liver 30 minutes after i.v. injection [69] and little hepatocyte transduction is observed due to sequestration of Ad5 by Kupffer cells and LSECs. This sequestration can be overwhelmed with higher doses [115], but this also increases toxic side effects. An alternate approach is to "predose" the system by injecting gadolinium chloride, chlodronate liposomes, or high doses of Ad5 to saturate and kill Kupffer cells before injecting the therapeutic or reporter virus [74, 116-120]. For example, predosing mice with Ad5 before injection of Ad5-luciferase increases hepatocyte transduction 44-fold [120]). Therefore, by eliminating Kupffer cells (and likely other cells like LSECs) with a first dose of Ad5, more functional virus is available to reach distant sites. In this case, when using a hepatocyte-tropic Ad5 the next downstream functional targets are hepatocytes that are effectively transduced by the virus. When performing oncolytic therapy against distant tumors, predosing increases the ability to kill tumors systemically [120]. Manickan *et al.* showed that in sequestering Ad5 virions, Kupffer cells themselves are destroyed [75]. Therefore, while liberated virions can escape for more distant delivery, this process is highly inflammatory and Kupffer cell evasion, rather than destruction, may be a preferable method [75, 121]. 

To what degree other Ad serotypes are trapped in the liver is still under investigation. Despite their close homology, preliminary comparison of species B Ad11 and 35, species C Ad5 and 6, and species D Ad26 and 48 for predosing demonstrated that Ad5 was by far the most effective at enabling a subsequent dose of Ad5-luciferase to transduce hepatocytes ([122] and data not shown). While this suggests that other Ad species may not be adsorbed by Kupffer cells, these other serotypes may be encountering sinks of their own, since they are not entirely neutral and some actually cause *lower *subsequent expression in hepatocytes. Closer examination of Ad6 suggests that it may evade Kupffer cells much better than Ad5 [14, 94]. Indeed, Ad5 and Ad6 are substantially better at liver-directed gene therapy than their family members Ad1 and 2 [14]. 

PEGylation of Ad also appears to be an effective means to detarget Kupffer cells. Random conjugation of succinimide-activated NHS-5 kDa PEG to Ad5 mediated marked reductions in Kupffer cell uptake in mice [70]. Interestingly, while this reduced many side effects (IL-6, thrombocytopenia, etc.), PEGylation did not appear to reduce uptake of virus into splenic cells (unpublished observations). Follow up testing of this approach with replication competent oncolytic Ad5 with different-sized NHS-PEGs (5, 20, and 35 kDa NHS-PEG) showed that 5 kDa PEG increased hepatocyte transduction, presumably by detargeting Kupffer cells [88, 107]. In contrast, larger PEG appeared to detarget both Kupffer cells and hepatocytes as evidenced by reduced liver expression. One possible cause for this reduction may lie in the failure of virions to access hepatocytes via fenestrae, due to the increase in virion diameter upon PEGylation. This dramatic increase in viral expression is not dependent on complete coating of the viral capsids, but could be replicated with specific conjugation of 5 kDa PEG into only the HVR5 loop of Ad5 hexon [114]. Subsequent systematic targeted PEGylation of HVR1, 2, 3, 4, 5, 6, and 7 in Ad5 demonstrated that shielding HVR1, 2 and 5 produced up to 20 fold increases in hepatocyte transduction whereas modification of the other HVRs had less effect [95]. Given that Kupffer cells are thought to phagocytose Ad5 via scavenger receptors, shielding the multiple negative charges particularly in HVR1 with this hydrophilic polymer likely mediates much of the protective effects on the virus.

### Evading the Liver: Hepatocytes

Based on the Ad5 prototype in mice, it appears that much of intravenously injected virus is sequestered by Kupffer cells and possibly by LSECs. Some fraction of any injected dose can escape these cells to go on to transduce hepatocytes or go further to infect extra-hepatic tissues. Kupffer cell depletion or evasion allows Ad5 virus to enter the next viral sink, which for this hepatotropic virus are hepatocytes, which yields productive transgene expression. If another Ad serotype is used that is less hepatotropic (e.g. due to lack of FX binding), then hepatocytes may not be the next viral sink, but it may be another tissue downstream. It appears that approximately 50% of human Ad serotypes do not bind FX [47], so these may be good platforms for hepatocyte evasion provided they do not infect the cells by other mechanisms.

Gene delivery by Ad in the liver occurs because of productive expression in hepatocytes [75, 83, 116-119, 123]. One method of hepatocyte detargeting uses pharmacologic ablation. In this case, warfarin can be used to inactivate vitamin K-dependent blood clotting factors and reduce Ad5 delivery to hepatocytes [120, 124]. While this is feasible in mice, achieving such low levels of FX in humans would likely be prohibitive given its effects on clotting. Modeling based on cryo-EM suggested that FX may interact with hypervariable (HVR) loops 3, 5 and 7 on Ad5 [125]. Similarly, insertion of peptides such as the biotin acceptor peptide (BAP) into HVR5 of replication competent Ad5 has demonstrated reduced expression of the luciferase transgene and increased expression in orthotopic tumors, thus extending survival [126].

While polymer modification of Ad may be expected to block Ad5-FX interaction, PEGylation appears to block Ad5 liver transduction directly. Indeed, random NHS PEGylation and targeted maleimide PEGylation both appear to preserve the ability of FX to bind to Ad5 virions [88, 95]. Given that RGD-integrin interactions appear functional on hepatocytes [68] and that integrin interactions are preserved after PEGylation [70], this interaction may be involved in maintaining hepatocyte transduction in the case of small PEGs. In contrast, large PEGs (20 kDa, etc.) may make the virus too big to cross fenestrae and/or use integrin associations, and therefore they may be used as a means to detarget hepatocytes.

### Evading the Liver: LSECs

Kupffer cells are thought to be the biggest sink for Ad5 in the liver, depletion of them by predosing surprisingly does not reduce the number of vector genomes in the liver [120, 127]. Combined predosing and warfarin improve oncolytic killing of distant tumors after i.v. injection, but these two detargeting strategies nevertheless do not significantly decrease viral genomes in the liver or increase viral genomes in the tumor at short time points [120]. In contrast, when Koski *et al.* treated mice with warfarin to deplete vitamin K dependent blood factor interactions, anti-platelet antibodies, and Kupffer cell scavenger receptor blockers into mice prior to Ad injection, the combination of these treatments yielded an 81% increase in tumor to liver ratio of virus [128].

These data suggest that virus may be sequestered by other cells of the liver. To address this, Shayakhmetov's group tested Kupffer and hepatocyte detargeting strategies combined with integrin detargeting by ablation of the RGD motif in Ad5 [68]. They showed that no single intervention by itself fully detargeted the virus from the liver. Rather, only when all three interventions were applied were significant reductions in viral sequestration observed. In particular, ablation of integrin binding appeared to detarget LSECs and hepatocytes, emphasizing the roles of both Kupffer cells and LSECs in viral trapping. Therefore, detargeting all three cell types (and maybe more) appears important to liberate virus for systemic delivery. 

Since PEG and HPMA polymers tend to reduce protein-protein interactions, it is not surprising that coating Ads with these hydrophilic polymers also has effects on interactions with endothelial cells. For example, coating Ad5 with 5 kDa PEG reduces infection and activation of human endothelial cells *in vitro *[67]. *In vivo,* PEGylation of Ad5 also reduces interactions with liver LSECs as evidenced by reduced upregulation of E-selectin messenger RNA [67]. 

### Evading Other Cells and Tissues

Progress is being made to avoid Kupffer cells, LSECs, and hepatocytes in the liver. As this predominant adenoviral trap is avoided, it is likely that new ones will surface. Currently, the spleen appears to be the next biggest pharmacologic sink, at least in mice [70]. The spleen appears to trap viral genomes approximately 3 times more than the liver in terms of viral to host genomes. Again, this is specific activity per unit tissue, so total amount of expression and viral genomes is profoundly higher in the liver. 

Splenic uptake likely occurs due to entrapment on blood cells and direct capture by macrophages. For instance, after i.v injection in mice the weight of the spleen increases 200% over the following 7 days [67]. When Ad5 is injected i.v. into splenectomized mice, the level of virus in the blood increases approximately 300% [67]. In the absence of a spleen, innate immune responses are also reduced 25% suggesting roughly that one quarter of the response is generated or modulated in this organ. 

Beyond the spleen, the lung is a likely sink by direct infection and also by the curious effect Ad5 (and perhaps other serotypes) has on Kupffer cells. Ad5 kills Kupffer cells within minutes of uptake [75] and ~4 hours later, the dead Kupffer cell fragments are released into the circulation. Like most large aggregates, these Kupffer cells are filtered from the circulation by the lungs and Ad-infested Kupffer cell remnants can be found in pulmonary capillaries. To what degree Ad remains active for gene delivery remains to be determined, particularly when replication-competent instead of replication defective virus is used. Indeed, Smith *et al.* have shown that cirrhotic rats actually suffer from substantially higher lung toxicity after Ad dosing than normal animals [129]. Therefore, evading Kupffer cells altogether may reduce transfer of virus to sites like the lung.

## PROTECTING ADENOVIRUS FROM THE IMMUNE SYSTEM

Host sequestration, viral traps, and pre-existing immunity necessitate the use of vastly larger doses of intravenously delivered vector for liver-directed or systemic therapy. However, as Ad capsids are known to be potent immunogens, delivering high doses of any Ad serotype will likely provoke hemagglutination, the formation of immune-complexes, activation of complement, recruitment of immune cells, the rapid and wide release of proinflammatory cytokines, and thus vigorous tissue damage (discussed in our original review). Given that adenoviruses are infectious agents, humans have widespread pre-existing immunity to different Ad serotypes. Similarly, vector induced immunity upon use as a therapeutic agent can prevent its use in the same person more than once. Therefore, we discuss the effect of innate and adaptive immune responses against human and non-human Ad vectors. 

### Evading Neutralizing Antibodies and Immune Responses

Upon primary exposure to Ads, an immunocompetent host will generate innate immune responses within hours of infection and robust adaptive responses are generated over the course of two weeks. Therefore, Ad vector administration in the naïve host produces transgene expression that generally peaks within days of injection after which expression is eliminated within two weeks due to CTL responses against Ad, Ad-infected cells, and/or transgene epitopes [130]. 

Upon secondary exposure to an Ad serotype, memory T cells expand a population of CTL effector cells more rapidly than in the first exposure to swiftly eliminate Ad-transduced cells. Furthermore, capsid-directed antibodies can neutralize a large fraction of virions to drastically blunt the level of transgene expression that would normally occur. The degree of neutralization can depend on the site of administration. Intravenous injection of Ad exposes it to large concentrations of systemic antibodies that efficiently decrease transduction [131]. In contrast, if the virus is injected at surfaces with lower levels of persistent antibodies (i.e. nasally, orally, vaginally), the systemically-immune host may not neutralize the virus effectively [132]. Similarly, injection into an isolated tissue (i.e. a tumor) or into an immunologically-privileged site (i.e. the eye) can prevent or reduce antibody neutralization. 

The ability to cross-neutralize another Ad is directly dependent on the ability of the polyclonal Ad antibodies to bind to conserved epitopes on the Ad surface. For example, neutralizing antibodies generated against Ad5 are most effective at neutralizing Ad5, but can also partially neutralize Ads of the same species. T cell responses can be more broadly cross-reactive, since functional protein structures are less variable. For example, amino acids involved with hexon trimer structure and interactions with other capsomer proteins are conserved even between Ad species and are good targets for major histocompatibility complex (MHC) class I and class II. On the other hand, HVRs on hexon are not intrinsic to function and widely vary to evade other serotypes’ neutralizing antibodies. 

As the most abundant capsid protein, most neutralizing antibodies are directed at hexon. Hexon-targeted antibodies appear to neutralize the virus not by blocking viral entry but instead by blocking microtubule transport of the virus to the nucleus after it escapes endosomes [133]. The second most prevalent neutralizing antibodies are against Ad fiber [54, 134]. These can block interactions with receptors or by targeting the virus to antigen-presenting cells [135]. Immune system targeting of these two proteins is also reflected Ad fiber and hexon protein diversity since a comparison of Ad serotypes shows that hexon and fiber are most variable even within one species of the virus [13, 37].

Multiple studies have shown that most humans are immune to Ad5, although levels of immunity are geographically variable and differences in testing for neutralization does not lend itself to ideal comparison. According to some estimates, 30-50% of Americans, 60% of Japanese, and 45-75% of people from Europe (Italy, UK, Netherlands, and Belgium) have pre-existing immunity to Ad5 [136, 137]. Ad5 seroprevalence can even reach as high as 100% of people in regions such as Brazil, sub-Saharan Africa and India [22, 138-140]. 

Ad seroprevalence within populations is strongly correlated with increasing age, presumably as a result of natural infections [139, 141]. One exception is infants below the age of 6 months, who can receive passive but transient immunity against Ad5 through maternally derived antibodies [139]**.** This seroprevalence data presents a clear “window” of low anti-Ad immunity in children between the age of 6 months and 7 years, and may signify the ideal time for Ad based therapies. 

Delivery of an Ad in a pre-immune host may not only attenuate therapy, but also have unexpected effects. A recent example is the now infamous Merck STEP HIV Vaccine trial. In this Phase II clinical trial, first generation (FG) E1-deleted Ad5 vaccines expressing gag, pol, and nef were used in individuals at high risk of HIV-1 infection as a T cell generating HIV vaccine strategy [142]. While this trial showed some positive immunologic effects, it was halted after failing to decrease HIV infection. Unexpectedly, early data from the STEP trial suggested that uncircumcised individuals with higher titers of pre-existing antibodies against Ad5 also had higher rates of HIV acquisition than volunteers with low Ad5 antibodies [142-144]. Based on this, Ad5-based vaccines fell out of favor [145-147]. 

Tests to understand STEP trial effect suggested that prior immunity to Ad5 followed by exposure to Ad5 could induce stimulate a population of CD4+ T cells that might become substrate for HIV infection [148]. However, further investigation has failed to demonstrate any true correlation between pre-existing immunity and likelihood of HIV infection [140, 149, 150]. Follow up of STEP vaccinees shows that there is no longer a statistical difference in HIV acquisition between groups with high or low antibodies vs. Ad [142]. Prior immunity to Ad5 in a parallel Ad5 trial called the Phambili trial had no effect on HIV acquisition [142]. Finally, a recent case control study shows that immunity to Ad5 does not pose increased risk of HIV infection [140]. 

Therefore, it is now unclear if the STEP trial effect was real or was a transient effect. Other Ad vaccines, such as the live attenuated Ad4 and Ad7 vaccines for military recruits are considered so safe that they are again in production as replication-competent vaccines [151].

### Adenovirus Serotype-switching to Evade the Immune System

One approach to evade neutralizing antibodies is to “serotype switch” the vector by changing the Ad serotype carrying genes with each administration [152]. This approach markedly increases prime-boost with Ad vaccines as demonstrated for HIV vaccines [21, 153-155]. Because of their low cross-reactivity and seroprevalence in humans, chimpanzee adenoviruses Ad-C68, 6, and 7 have been studied for vaccine purposes. Ad-C68 was shown to be effective at inducing anti-rabies neutralizing antibodies and may be capable of inducing anti-HIV-1 gag CTL immune responses [21, 156]. Santra *et al.* confirmed that simian Ads C7, C68 and chimeric C1/C5 were capable of inducing immune responses in the presence of pre-existing immunity and could be used in prime/boost immunization strategies [157]. In addition to their use as vaccine vectors a simian adenovirus ChAd3 was shown to be effective at expressing carcinoembryonic antigen (CEA) and was as robust as huAd5 at breaking tolerance and successfully overcoming tumorigenicity in the presence of huAd5 pre-existing immunity [158].

Liu *et al.* explored the use of a recombinant human serotype 26 adenovirus as a vector for a T-cell based anti-SIV vaccine. A further advantage of Ad26 was that it was only found to have a 21% level of seroprevalence in Sub-Saharan Africa, a region in desperate need of an HIV-1 vaccine [22]. Using a heterologous prime/boost of Ad26 and Ad5 expressing SIV gag they were able to show a 1.4 and 2.4 log reduction in peak and chronic viremia levels, respectively [159]. Ad48 was found to have a seroprevalence of only 3% in the same region [22]. In another study, the hexon hypervariable regions (HVRs) of Ad5 were replaced with the HVRs of Ad48 hexon. This new Ad5HVR48 virus expressed SIV Gag, Pol, Nef and Env and immunized macaques were found to have lower peak and setpoint viremia levels [160]. 

The vast majority of work with adenovirus vectors has utilized first generation Ad (FG-Ad) vectors that are typically deleted for their E1 and E3 genes (see [12]). FG-Ad vectors carry 17 Ad protein open reading frames (ORFs) that can be expressed and targeted by anti-Ad T cell responses [161, 162]. In contrast, in helper-dependent adenoviral (HD-Ad) vectors, all viral ORFs are deleted [163-165]. No Ad proteins are produced in HD-Ad vector-transduced cells thereby evading T cells responses that can kill transduced cells [163-165]. This reduced immunogenicity and reduced liver damage allows for transgene expression in mice and in baboons over years [8, 166-168]. The HD-Ad system is also uniquely suited to serotype switching, since Ads of the species can cross-package each other’s genomes. HD-Ads therefore give the opportunity to evade both anti-Ad T cell responses and Ad neutralizing antibodies. Their low immunogenicity not only increases their safety, but also increases their persistence *in vivo*. We recently studied the utility of serotype switching HA-Ads 1, 2, 5 and 6 that expressed HIV Env gp140 [23] to show that serotype switching in both mice and rhesus macaques boosted anti-HIV immune responses. A subsequent challenge of the HD-Ad serotype switch immunized macaques resulted in impressive immune control of viremia in SHIV-SF162P3 challenged animals with 2 – 10 fold decreases in peak viremia with set-point viremia levels ~4 logs lower [169].

### Polymer Modification of Ads to Evade Immune Responses

An original attraction of polymers like PEG for Ad coating was to protect it from neutralizing antibodies [98, 101, 102]. When tested in mouse models, PEG and HPMA polymers are able to protect Ad from pre-existing and vector-induced neutralizing antibodies to allow multiple administrations into immune recipients [93, 98, 101, 102]. Ad PEGylation also reduces the production of new antibody and cellular immune responses against Ad proteins [101]. 

While PEGylation does cover the virus with as many as 15,000 PEGs, it does not completely shield the virus and PEGylated vectors recover only 10% of normal vaccine activity in the face of anti-Ad antibodies ([93] and data not shown). In addition, the negative effects of random PEGylation on virus transduction can mitigate the benefits of antibody shielding. For example, when we tested succinimide-activated NHS-PEG for gene-based vaccination with Ad, this random PEGylation ablates CAR-mediated transduction *in vitro* and reduces *in vivo* transduction after intramuscular (i.m.) and intranasal (i.n.) vaccination by 50 to 90% [93]. This loss of activity by i.m. and i.n. routes differs markedly with the retention of *in vivo *transduction after i.v. injection [70, 88, 107]. This difference is likely due to loss of needed CAR binding by i.m. and i.n. transduction as compared to FX-mediated transduction after i.v. injection. 

One possible solution to this conundrum is the use of targeted PEGylation of Ad [95, 105, 106, 114]. These targeted PEG vectors not only retain full *in vitro* transduction, but targeted PEGylation of Ad at HVR1, 2, 5, and 7 actually increases transduction after i.v. injection up to 20-fold [95]. To what degree polymer modification will protect viruses from antibodies *in vivo* remains to be demonstrated in prime-boost systems.

While there is some question of exactly how well polymers can protect virus from neutralizing antibodies, there is good evidence showing that PEGylation can blunt many of the innate immune responses against Ad [70, 170-172]. When injected i.v. into mice, PEGylation reduced innate immune responses as evidenced by a 90% reduction in IL-6 over 48 hours [70] and IL-12 and TNF-alpha levels were reduced three- and seven-fold, respectively [170]. In baboons, PEGylation reduced IL-6 3-fold, IL-12 by 50% [172]. Notably, PEGylation of Ad also reduces uptake of the virus into antigen-presenting cells including macrophages and Kupffer cells [70]. It also inhibits complement activation by Ad5 [58]. 

## ADENOVIRAL VECTOR RETARGETING 

A better understanding of the sinks that absorb adenoviruses is crucial to retargeting these vectors to novel receptors and increasing its therapeutic success. Our earlier review and several others have discussed a number of strategies for vector retargeting which is crucial for both targeted gene therapy and oncolytic purposes [12, 173-175]. Below, we highlight new areas of Ad retargeting approaches that have been applied since the last review.

### Peptide Inserts/Ligands/Linkers

The selection of targeting peptides from phage libraries and their insertion into the viral capsid has been reviewed in previously [12, 176]. These methods were unpredictable with regard to whether peptide insertions would be tolerated by the virus and retain their specificity. One approach to circumvent these problems involved selecting peptides from a bacteriophage library that displayed random peptides in the context of the Ad fiber HI loop, into which it would later be cloned [177]. More recently, Ad peptide libraries have been created in which random peptides are cloned directly into the Ad capsid. In this way, functional virions can be directly selected [178-180]. This technique was modified to accommodate the insertion of peptides with known affinity for cellular targets. Lupold *et al.* designed an Ad peptide library that had a constant binding peptide insert flanked with random linker sequences [179]. Virions could then be selected for functional virus with retained binding specificity [181]. Although these Ad libraries could theoretically contain up to 10^9^ unique peptides, current techniques to produce actual Ad virions have only yielded library sizes of up to 2 x 10^5 ^[178, 179]. This imposes a significant restriction of the size of peptides that can be screened as ligands. For example, a 10^5 ^peptide library in Ad can represent only four amino acid-long random peptides. In contrast, a 10^10^ peptide library on bacteriophage can represent seven amino acid peptides (reviewed in [176]).

The success of peptide insertions for targeting is not only dependent on the peptides, but also on their location on the capsid. Ad peptide libraries are currently limited to fiber insertions. The generation of pIX or hexon based Ad peptide libraries may yield viruses that can take advantage of low affinity, high avidity interactions. The recent high resolution x-ray crystal and cryo-EM based structures of adenovirus may provide new insight into other regions of the virus that may tolerate modification for targeting [182-184]. 

Targeting adenovirus through the incorporation of high affinity proteins like antibodies has been hampered by both the large size of antibodies and improper folding of antibodies in the reducing environment of the nucleus where Ad is assembled. To circumvent this incompatibility, molecular adapters have been designed to bind to the Ad capsid outside of the context of the nucleus. The first such adapter consisted of a soluble form of CAR (sCAR) fused to targeting molecules like FGF-2, EGF [185], carcinoembryonic antigen (CEA)[186, 187], and folate [188, 189]. More recently, another targeting adapter molecule consisting of the GLA domain from FX fused to single-chain antibodies was developed [190]. The FX fusion protein binds to hexon, and therefore has 240 binding sites as opposed to the 12 available fiber proteins available to sCAR on the viral capsid. By binding to the HVR of hexon, native FX binding is reduced and enables detargeting from the liver. Another possible benefit of this technique is that it may allow improved spread of virus in an oncolytic setting since targeting is not affected by excess fiber production [190]. A major hurdle to this targeting method is that the adaptor molecules rely on non-covalent protein-protein interactions for their conjugation to the Ad capsid, which are generally considered too weak *in vivo. *Naturally occurring antibodies or CAR receptors could compete for Ad binding and displace the molecular adaptors from the capsid, abolishing the vector targeting activity.

In a third adapter molecule strategy, ankyrin repeat proteins (DARPins) have been designed to bind to fiber with low nanomolar affinity [191-193]. DARPins are cysteine-less alternatives to antibodies that consist of helical repeats containing protein interaction surfaces. A DARPin library was created and used to select DARPins specific for both fiber and the target protein HER2. Fusion molecules of fiber/HER2 binding DARPins can be produced in *E. coli* and have been used to target adenovirus to HER2 positive cells. Further study will be needed to determine if these adapter proteins will have any utility *in vivo *[194]. 

## SUMMARY

As research in Ad biology progresses, we begin to appreciate that vector pharmacology is less reliant on direct receptor binding and more influenced by complex interactions between virus and host. Ad engineering efforts have generally been concerned with targeting vectors to an intended location, and recent research demonstrates the paradigm shift from fiber to hexon modification. *In vivo*, the interactions of hexon and blood factors can be harnessed for liver-directed gene therapy. Conversely, this same interaction can be a considerable viral sink for the purposes of therapy beyond the liver. 

Our growing understanding of Ad biology suggests that Ad encounters progressive viral sinks in addition to blood factors, such as interactions with circulating cells, antibodies, and complement. More formidably, organs like the liver trap enormous doses of therapeutic vector particles, yet even this sequestration is partitioned into regions such as hepatocytes, Kupffer, and endothelial cells. Here we summarize not only the obstacles Ads face *in vivo*, but also strategies that have been used to evade them. It has become clear that direct targeting strategies may have significantly increased success when detargeting strategies are applied in combination.

## Figures and Tables

**Table 1:** Classification of Human Adenoviral Serotypes [37, 195]

Species	Serotype
A	12, 18, 31
B	3, 7, 11, 14, 16, 21, 34, 35, 50, 55
C	1, 2, 5, 6
D	8, 9, 10, 13, 15, 17, 19, 20, 22, 23, 24, 25, 26, 27, 28, 29, 30, 32, 33, 36, 37, 38, 39, 42, 43, 44, 45, 46, 47, 48, 49, 51, 53, 54
E	4
F	40, 41
G	52

**Table 2:** Summary of Encapsidated Proteins in Adenovirus Serotype 5 [2, 33, 196-198]

Protein Number	Protein Name	Size (kDa)	Number Per Virion	Known Functions
II	Hexon monomer	110	720	Structural; liver tropism
III	Penton base	63	60	Structural; binds cellular integrins
IIIa	Cement protein	63	60	Associated with penton base
IV	Fiber	62	36	Primary attachment protein
V	Core protein	42	160	Associates with DNA and penton to connect the nucleocore and capsid
VI	Cement protein	23	~360	Endosomal lysis and escape; imports hexon into the nucleus for viral assembly
VII	Core protein	19	840	Histone-like
VIII	Cement protein	15	120	Associated with underside of hexon capsid; Stabilization/assembly of particle?
IX	Cement protein	14	240	Stabilization/assembly of capsid
TP	Terminal Protein	55	2	Protein primer for genome replication
X	Mu	4	100	Nucleoprotein; Genome replication?
IV2a	Nucleoprotein			Genome packaging
	Protease	23	~10-12	Viral protein processing and maturation
